# Coexistence of Antibiotic Resistance Genes and Virulence Factors Deciphered by Large-Scale Complete Genome Analysis

**DOI:** 10.1128/mSystems.00821-19

**Published:** 2020-06-02

**Authors:** Yu Pan, Jiaxiong Zeng, Liguan Li, Jintao Yang, Ziyun Tang, Wenguang Xiong, Yafei Li, Sheng Chen, Zhenling Zeng

**Affiliations:** a Guangdong Provincial Key Laboratory of Veterinary Drugs Development and Safety Evaluation, South China Agricultural University, Guangzhou, China; b National Risk Assessment Laboratory for Antimicrobial Resistance of Animal Original Bacteria, South China Agricultural University, Guangzhou, China; c Guangdong Laboratory for Lingnan Modern Agriculture, Guangzhou, China; d Department of Environmental Engineering, Technical University of Denmark, Kongens Lyngby, Denmark; e Public Monitoring Center for Agro-product of Guangdong Academy of Agricultural Sciences, Guangzhou, China; f Department of Infectious Diseases and Public Health, Jockey Club College of Veterinary Medicine and Life Sciences, City University of Hong Kong, Kowloon, Hong Kong, China; University of California San Diego

**Keywords:** antibiotic resistance, virulence factors, gene coexistence, bacterial genome, pathogens, natural environment, public health risk

## Abstract

Widespread use of antibiotics has enhanced the evolution of highly resilient pathogens and poses a severe risk to human health via coselection of antibiotic resistance genes (ARGs) and virulence factors (VFs). In this study, we rigorously evaluate the abundance relationship and physical linkage between ARGs and VFs by performing a comprehensive analysis of 9,070 bacterial genomes isolated from multiple species and hosts. The coexistence of ARGs and VFs was observed in bacteria across distinct phyla, pathogenicities, and habitats, especially among human-associated pathogens. The coexistence patterns of gene elements in different habitats and pathogenicity groups were similar, presumably due to frequent gene transfer. A shorter intergenic distance between mobile genetic elements and ARGs/VFs was detected in human/animal-associated bacteria, indicating a higher transfer potential. Increased accumulation of exogenous ARGs/VFs in human pathogens highlights the importance of gene acquisition in the evolution of human commensal bacteria. Overall, the findings provide insights into the genic features of combinations of ARG-VF and expand our understanding of ARG-VF coexistence in bacteria.

**IMPORTANCE** Antibiotic resistance has become a serious global health concern. Despite numerous case studies, a comprehensive analysis of ARG and VF coexistence in bacteria is lacking. In this study, we explore the coexistence profiles of ARGs and VFs in diverse categories of bacteria by using a high-resolution bioinformatics approach. We also provide compelling evidence of unique ARG-VF gene pairs coexisting in specific bacterial genomes and reveal the potential risk associated with the coexistence of ARGs and VFs in organisms in both clinical settings and environments.

## INTRODUCTION

Antibiotic resistance is widely recognized as one of the major crises of public health. It has led to decreased efficacy of antimicrobials in clinical practice and even a lack of effective drugs in treating infections caused by multidrug-resistant bacteria. In Europe, nearly 33,000 people die annually because of antibiotic resistance (1). More worryingly, the emergence of antibiotic-resistant bacteria that contain virulence factors (VFs; which enable a microorganism to establish itself on or within a host and enhance its potential to cause disease, include bacterial toxins, cell surface proteins that mediate bacterial attachment, cell surface carbohydrates and proteins that protect a bacterium, and hydrolytic enzymes that may contribute to the pathogenicity of the bacterium) has inevitably facilitated clinical outbreaks, causing life-threatening infections and serious public health threats (2, 3).

The virulence mechanisms in bacteria have been modified over millions of years to enhance their adaptation in host defense systems. However, the global emergence and development of antimicrobial resistance has only accelerated over the last 50 years, i.e., since antibiotics were first used. Although antibiotic resistance and bacterial virulence have developed in different timescales, there is likely an interplay (coselection) between antibiotic-resistant genes (ARGs) and VFs under selective pressure (4). In our experience, increased resistance has always been associated with decreased virulence and fitness in that interplay, either directly or indirectly (5). However, this may not be true in all cases. There is increasing evidence indicating that the relationship between resistance and virulence is adjusted in such a way that it confers more benefit to the pathogen (6, 7). Virulence factors are essential for bacteria to overcome host defense systems, and the acquirement of antibiotic resistance contributes to overcome antimicrobial therapies and to adapt to and colonize demanding environments (8). The associations between ARGs and VFs might follow the Darwinian model, in which those characteristics (e.g., antibiotic resistance plus virulence) that confer a specific advantage will be selected and fixed. Simultaneously, gene transfer events and a large genetic library can be utilized by bacteria to compensate for or overcome fitness costs, resulting in the constant emergence of resistant and virulent clones.

Widespread occurrence of ARGs and VFs in single clinical lineages or complexes of multiple pathogenic species has frequently been reported in recent decades, such as in Staphylococcus aureus (9), Klebsiella pneumoniae (3), Pseudomonas aeruginosa (10), *Enterococcus* spp. (11, 12), and Escherichia coli ST131 (13). ST11 carbapenem-resistant hypervirulent Klebsiella pneumoniae strains cause severe pneumonia with poor response to antibiotic treatment. Dissemination of such pathogenic strains is associated with a high mortality rate in Asia (3). In particular, methicillin-resistant Staphylococcus aureus with multidrug-resistant genes and virulence mechanisms continues to be associated with significant morbidity and mortality (>26%) in community infections (2). The coselection between ARGs and VFs has also been reported. Significant coexistence of ARGs and VFs was detected in Escherichia coli recovered from wastewater treatment processes (14). The coselection of ARGs and VFs located in mobile gene elements (MGEs) was also detected in Escherichia coli (15, 16). However, to our knowledge, previous studies on ARG-VF coexistence have lacked comprehensive data compilation that enabled us to detect common patterns of bacteria in gene selection and evolution processes. Hence, it is essential to systematically investigate and comprehensively understand the ARG-VF coexistence profiles among bacteria from different species and habitats.

In this study, to uncover coexistence profiles of ARGs and VFs in diverse bacterial categories, we used bioinformatic approaches to analyze the large-scale bacterial complete genomes available in the NCBI repository. We investigated the genetic relationships between ARGs and VFs across genome categories (phylogenies, pathogenicities, and habitats) from different perspectives and obtained the abundance profiles of broad-spectrum ARG/VF types and high-resolution evaluation of their genetic relationships. Furthermore, the underlying coexistence structures in different ecologies and the cotransfer potential of ARGs and VFs were also assessed. Comprehensive analysis of the coexistence profiles of ARGs and VFs in a large number of bacterial genomes would not only improve our understanding of the impact of genetic events in shaping the formation of the ARG and VF pool but also provide compelling evidence for the coexistence of unique ARG-VF combinations in specific bacterial genomes.

## RESULTS AND DISCUSSION

### Abundance and enrichment profiles of ARGs and VFs.

We randomly collected 9,070 bacterial complete genomes that covered a large diversity of phylogenies (see Fig. S1 in the supplemental material). The ARGs and VFs were identified in 76% (6,875 genomes in 24 phyla) and 50% (4,563 genomes in 8 phyla) of the 9,070 bacterial complete genomes, respectively. In total, 346 genomes (ca. 3.8%) had high abundances (>50 hits per genome) of both ARGs and VFs, including genomes of *Escherichia*, *Salmonella*, *Pseudomonas*, and *Shigella* strains. The propagation of ARGs in enteric microorganisms was prominent. All of the top 100 genomes that carried abundant ARGs belonged to *Enterobacteriaceae*, which were deemed as a potential ARG repository. The enrichment of ARGs in *Enterobacteria* was consistent with previous studies, which reported the continuous occurrence of typical ARGs in gastrointestinal microbial communities subjected to persistent antibiotic selection pressure (17–19). In contrast, VFs were more abundant in *Legionellaceae*, accounting for nearly 80% of the top 100 VF abundance genomes.

10.1128/mSystems.00821-19.1 FIG S1 ARGs and VFs in the complete genome dataset. The trees were generated to show the phylogenetic lineages of each strain using Cytoscape. The strain nodes (gray) are colored when they carry ARG or VF. Three main phyla (*Proteobacteria*, *Firmicutes*, and *Actinobacteria*) are separately highlighted as circular trees, and their ARG and VF abundance profiles (log_2_ transformed) are presented in the outer section. Download FIG S1, TIF file, 2.7 MB. Copyright © 2020 Pan et al. 2020 Pan et al. This content is distributed under the terms of the Creative Commons Attribution 4.0 International license.

To ascertain whether there was a significant correlation between the abundances of ARGs and VFs in specific phylogenies, the average ARG/VF abundances were counted across different phylogenies. Nearly half (23 genera) of the top 50 genera for ARG and VF abundance in data set were shared. Furthermore, *Enterobacteriaceae* occupied over 25% (the largest proportion) of the 23 intersecting genera. However, no significant linearity (*R*^2^ <0.5) was detected between ARG and VF abundance in *Enterobacteriaceae* (Fig. S2), indicating that there was no obvious joint enrichment between ARGs and VFs in *Enterobacteriaceae*, although the coexistence phenomenon was apparent in some strains of *Enterobacteriaceae*. *Enterobacteriaceae*, which usually act as commensals in various hosts, will be hampered by a short survival time in the host due to lethality caused by the excessive coaccumulation of resistance and virulence genes. Notably, the *mcr*, *bla*_NDM_, and *tet*(X) genes, which encode resistance to last-resort antibiotics (colistin, carbapenem, and tigecycline), could be detected in *Enterobacteriaceae*, as could VFs. Just as the frequent outbreaks of pathogenic and resistant Klebsiella pneumoniae (3), Escherichia coli (20), and Salmonella enterica (21) have been devastating, *Enterobacteriaceae*, which may be elicited by selective pressures, pose a large threat to public health and may cause antibiotic failure during clinical infections. This is contributed by the high abundance of ARGs and VFs and resistance to last-resort antibiotics. The coexistence of a high abundance of ARGs and VFs in *Enterobacteriaceae* indicates that modern anthropogenic activities could drive and shape bacterial genic selection, although virulence and resistance have evolved over very different timescales (22). While large-scale complete genome analyses could demonstrate a comprehensive profile of ARGs and VFs at the forefront of our current knowledge, possible biases may exist in the data due to the uneven sources in public databases and the lack of accessible biological information of largely unculturable environmental microbes (Fig. S3).

10.1128/mSystems.00821-19.2 FIG S2 ARG and VF abundance correlation in *Enterobacteriaceae*. The ARG and VF abundance correlation in *Enterobacteriaceae* is nonsignificant (*R*^2^ < 0.5). Download FIG S2, TIF file, 0.9 MB. Copyright © 2020 Pan et al. 2020 Pan et al. This content is distributed under the terms of the Creative Commons Attribution 4.0 International license.

10.1128/mSystems.00821-19.3 FIG S3 Composition of the complete genome collection in different groups. (a) phyla; (b) habitats; (c) pathogenicity. Download FIG S3, TIF file, 2.6 MB. Copyright © 2020 Pan et al. 2020 Pan et al. This content is distributed under the terms of the Creative Commons Attribution 4.0 International license.

In the broad-scale gene detection, an uneven abundance in the distribution of ARG and VF types was revealed (Fig. S4). The top five dominant ARG types (excluding multidrug) were beta-lactam (7.24%), aminoglycoside (6.24%), bacitracin (6.12%), macrolide-lincosamide-streptogramin (MLS) (5.92%), and polymyxin (5.72%). Among the 13 VF types we retrieved, secretion system, adherence, metal uptake, and toxins accounted for more than 80% of the total VF numbers. Antibiotic consumption has been recognized as the main cause of ARG emergence. The ARGs for commonly consumed antimicrobials were more likely to be highly abundant in this study, which was also consistent with previous abundance-based surveys (23, 24).

10.1128/mSystems.00821-19.4 FIG S4 ARG and VF abundance in different gene types across complete genomes. Download FIG S4, TIF file, 0.3 MB. Copyright © 2020 Pan et al. 2020 Pan et al. This content is distributed under the terms of the Creative Commons Attribution 4.0 International license.

Next, ARG and VF enrichment in different genomes was evaluated (Fig. S5). Among the four habitats under investigation, most of the ARG types were more likely to be enriched in the human, animal, and soil habitats (*P* < 0.01 and odds ratio [OR] > 1 [Fisher exact test with Benjamini-Hochberg correction]). Across these four major phyla, enrichment was observed in 24 ARG types. We found a higher diversity of ARGs enriched in pathogens than nonpathogens, which was consistent with the results of a previous analysis (25). Among VFs, all 13 types were enriched in different phyla, and more tended to be enriched in the human, animal and soil habitats. The enrichment of “advantageous” genes may protect microorganisms from the detrimental conditions brought by environment and host (26). To summarize, these results indicate a situation of broad-spectrum antimicrobial resistance and virulence disseminating in human pathogens and animal-associated bacteria.

10.1128/mSystems.00821-19.5 FIG S5 Binary heatmap of ARG (a) and VF (b) enrichment by phylogeny, habitat, and pathogenicity. The cells of the heatmap are colored in blue if the ARG or VF type is significantly enriched in a particular phylogeny, habitat or pathogenicity (*P* < 0.01 and OR > 1 [Fisher exact test]). Download FIG S5, TIF file, 0.4 MB. Copyright © 2020 Pan et al. 2020 Pan et al. This content is distributed under the terms of the Creative Commons Attribution 4.0 International license.

### Coexistence of ARGs and VFs.

**FIG 1:**
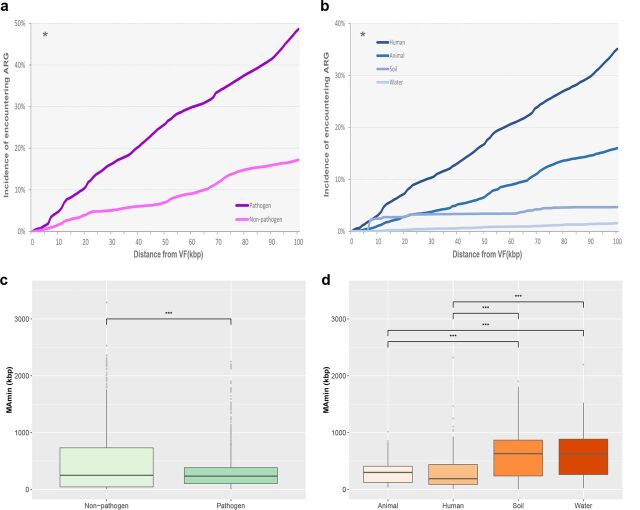
ARG and VF coexistence by pathogenicity (a and b) and habitat (c and d). (a and c) Incidence of encountering ARGs along the distance from VFs (*, *P* < 0.05 [determined by Fisher exact test]); (b and d) MetAmin index (***, *P* < 0.001 [determined by Student *t* test]).

The coexistence profiles of ARGs and VFs were compared among organisms that belonged to different pathogenicity groups (containing pathogens and nonpathogens; the pathogen group consisted of all the confirmed and potential pathogens) and that resided in different habitats (Fig. 1). The coexistence trend was more significantly confirmed in pathogens than nonpathogens. The encounter incidence of ARGs in pathogen genomes (49% at 100 kbp) was always higher than that in nonpathogen genomes (17% at 100 kbp) as the distance from the VFs increased, indicating that VFs have a close connection with the occurrence of ARGs in pathogens (*P* < 0.05, Fisher exact test). In the MetAmin profile, a significantly closer distance between ARGs and VFs was observed in genomes with potential pathogenicity (*P* < 0.001, Student *t* test), which further strengthened the previous argument. Among the habitat groups, human-associated bacteria had the highest encounter incidence of ARGs, while animal-associated bacteria had the smallest MetAmin. When antibiotics began to be widely used in clinical treatment after the 1940s, the evolution of pathogens was greatly affected by the indiscriminate use of antibiotics. The development of ARGs and VFs in pathogens overlapped considerably, and it was not possible to consider them as separated processes (27). In recent years, bacteria that were resistant to many antimicrobials and carried virulence factors, such as Enterococcus faecium (CC17), Streptococcus pneumoniae (PMEN1) and E. coli ST131 (28–30), had spread globally. These clones were considered highly successful or high-risk strains and could be detected in both humans and animals (31, 32).

To ascertain which pathogen species contributed to the above coexistence phenomenon, a profile of the 31 pathogen species with high coexistence potential was assessed (Fig. S6). Of the 31 high-coexistence-potential species, 14 exhibited a higher incidence of encountering ARGs, along with an increase in the distance from VFs, particularly in Salmonella bongori, Citrobacter freundii, Fusobacterium nucleatum, Pseudomonas aeruginosa, and Escherichia coli. Moreover, the MetAmin profile revealed substantial coexistence potential among the above 14 pathogen species, since their average MetAmin value (184 kbp) was much shorter than that of nonpathogen genomes (463 kbp). Upon examining the coexistence profiles in different habitats, the coexistence signature was more apparent in genomes derived from human and animal habitats, but it was much less frequent in organisms in soil and water habitats. This observation might be related to a phenomenon whereby pathogens in human and animal habitats are regularly exposed to antimicrobial agents and experience strong selection pressure (33).

10.1128/mSystems.00821-19.6 FIG S6 ARG and VF coexistence in 31 pathogen species with ARG and VF coselection potential. (a) Incidence of encountering ARGs along the distance from VFs (14 species that have higher incidence are colored); (b) MetAmin value in the corresponding 31 species. Download FIG S6, TIF file, 1.4 MB. Copyright © 2020 Pan et al. 2020 Pan et al. This content is distributed under the terms of the Creative Commons Attribution 4.0 International license.

### Diversity of nearest ARG from VFs.

**FIG 2:**
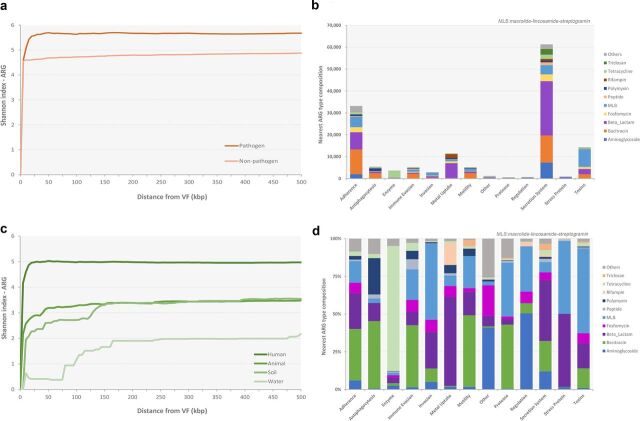
ARG diversity profile along the distance from VFs. Shannon diversity scores of the ARG subtypes along the distance from VFs by pathogenicity (a) and habitat (c), the abundance profile of the nearest ARG from all VF types (excluding multidrug) (b), and the composition of the nearest ARG from all VF types (excluding multidrug) (d) are shown.

Although the correlation of ARGs and VFs has been sporadically reported (19, 27, 34–36), there is still a lack of systematic knowledge of the general diversity pattern in bacterial genomes. In this study, the coexistence diversity of ARGs was found to be different between genomes of organisms in diverse pathogenicity groups and habitats, but there was no clear difference between animal and soil habitats (Fig. 2a and c). Furthermore, a higher Shannon diversity of ARG was detected in bacteria in the human habitat and pathogen category, suggesting that VFs cooccur with a large-spectrum of ARGs in human-associated pathogens. During the process of bacterial evolution, conserved genes are not uniformly distributed but are organized into clusters. It was suggested in previous studies that gene couples have a close functional relationship if the gene clusters exhibit the same coevolution pattern across different genomes (37). To identify ARG-VF couples that tend to be functionally related, the closest ARGs for all VFs were counted by type (Fig. 2b). The top five abundant ARG types (excluding multidrug), which are most likely to be functionally related to VFs and potentially confer resistance to major classes of antibiotics, encompass beta-lactam, bacitracin, MLS, fosfomycin, and aminoglycoside. In addition, different VF types were commonly present with distinct ARG types as their nearest genetic neighbor (Fig. 2d). For example, enzymes were more likely to correlate with tetracycline; stress proteins with MLS and beta-lactam; and antiphagocytosis with bacitracin, polymyxin, and beta-lactam.

### Clusters of coexistence structures in different genome categories.

The composite structure of the ARG-VF pairs was evaluated using principal coordinate analysis (PCoA) to identify whether there was a significant bacterial genome clustering pattern among different genome categories (Fig. S7). Intriguingly, a nonsignificant clustering was detected (*P* > 0.001, analysis of similarities [ANOSIM]) in the genomes from different habitats and pathogenicities. Even if genomes in different ecologies had individual ARG and VF coexistence profiles, we should bear in mind that intense gene dissemination may promote the acquisition and spread of the ARGs and VFs. The nonsignificant clustering suggested that the spread of VF-associated ARGs in bacteria was frequent enough to blur the resistome boundaries in different pathogenicities and habitats. Antibiotic resistance existed before the discovery and invention of currently used antibiotics, and the majority of ARGs may have an environmental origin (38). The high taxonomic and genetic diversity in environments (e.g., soil and sewage) has led to a nonuniform abundance and diversity of ARGs profiles, which are only partially governed by selection pressure. Moreover, the specific bacterial taxa that thrive in several different habitats are likely to play an important role and might serve as mediators for ARGs in crossing ecological dissemination barriers (39–41). Another possible driving force to transmit ARGs across habitats is the increasingly prominent anthropogenic pressure, such as municipal wastewater systems, pharmaceutical manufacturing effluents, aquaculture facilities, and animal husbandry facilities (22). These sites are characterized by extremely high microbial loads coupled with subtherapeutic concentrations of antimicrobials, contributing to the discharge of human/animal fecal microbes and antibiotic-resistant organisms into the environment (42). Globally, the nonpathogenic species and commensals, regardless of their ecological niche, are now less susceptible to antibiotics than before (43). ARG dissemination between pathogens and nonpathogens was also observed. Due to horizontal gene transfer (HGT), pathogens under strong selective pressure may undergo positive selection of favorable genes (e.g., ARGs and VFs) originally belonging to nonpathogens to enhance their fitness (44). In addition, the core ARG-VF coupling types were searched across all the genomes. However, there was no single ARG-VF pair shared among all the genomes. There were five coupling types (multidrug with adherence, secretion system, metal uptake, bacitracin with adherence, and beta-lactam with metal uptake) identified in more than 25% of genomes, which mainly included couples of multidrug ARGs and VFs, highlighting the diversity of the ARG-VF alliance.

10.1128/mSystems.00821-19.7 FIG S7 PCoA plots of ARG and VF coexistence structure in genomes by pathogenicity (a) and habitat (b). Genomes of different habitats and pathogenicity status do not cluster significantly (*P* > 0.001 [ANOSIM]). Download FIG S7, TIF file, 2.6 MB. Copyright © 2020 Pan et al. 2020 Pan et al. This content is distributed under the terms of the Creative Commons Attribution 4.0 International license.

**FIG 3:**
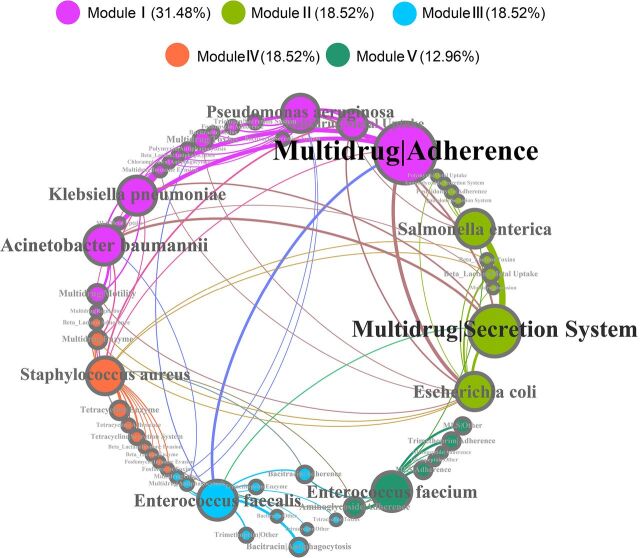
Network (circular layout) revealing the coexistence patterns between ARG-VF pairs and major pathogen species. The nodes are colored according to modularity class. The connections between each node represent that the relative abundance of ARG-VF pairs in that pathogen species exceeds 0.01. The size of each node is proportional to the weighted number of connections, that is, the average weighted degree.

In the coexistence network analysis, a comprehensive perspective of the connection between ARG-VF pairs and important pathogen species was demonstrated (Fig. 3). Not surprisingly, the majority of these detected broad-spectrum ARG-VF pairs were shared between different pathogen species. The frequent spread of VF-associated ARGs, which might cross ecological dissemination barriers, was further confirmed. Based on the modular classification, there was a significant modular structure (i.e., clusters of nodes that interact among themselves more than other nodes in a random association) observed in the network (the modularity index was 0.465) (45). Every module had a distinct coexistence constitution and a more frequent exchange of ARG-VF pairs between species in the module. Module I, containing Klebsiella pneumoniae, Pseudomonas aeruginosa, and Acinetobacter baumannii, occupied a large number of vertices. These pathogen species contained a higher diversity of ARG-VF pairs and had a closer correlation in ARG-VF coexistence. Moreover, an apparent luxuriance of multidrug-adherence and multidrug-metal uptake gene type pairs was detected in module I. Module II, containing Salmonella enterica and Escherichia coli, was more likely to carry gene type pairs that contained multidrug and beta-lactam resistance genes, such as multidrug-secretion system and beta-lactam-metal uptake. Module III (containing Enterococcus faecalis), module IV (containing Staphylococcus aureus), and module V (containing Enterococcus faecium) frequently carried the ARG-VF pairs containing bacitracin, multidrug and tetracycline, and MLS and aminoglycoside resistance genes, respectively. Pathogen species in the same module might reside in similar environmental niches and under common selection pressures. Positive selection in pathogenic species to different ARG-VF pairs could provide a functional support for their survival or development in specific niches.

### Transfer potential of ARGs and VFs.

**FIG 4:**
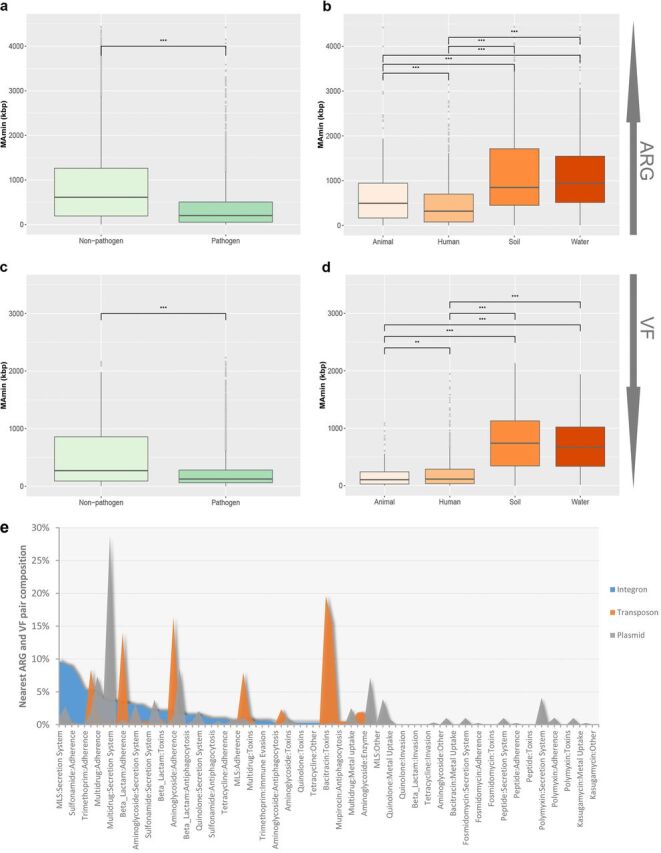
ARG and VF cotransfer potential. (a to d) The MetAmin index boxplot of the nearest ARG (a and b) and VF (c and d) from each MGE in each genome across the different genome categories (***, *P* < 0.001; **, *P* < 0.01 [determined by Student *t* test]); (e) cotransfer preference of the three MGE types (transposon, integron, and plasmid) for the nearest ARG and VF pair across the data set.

The cotransfer ability of ARGs and VFs in different pathogenicities and habitats was evaluated based on the nearest distance from MGEs (Fig. 4). The distances of MGEs from the nearest ARGs and VFs were much shorter in the genomes from pathogen species and human and animal habitats (*P* < 0.001, Student *t* test). The shorter distances suggested a possible role for MGEs in transferring antibiotic resistance and virulence in human pathogens and animal-associated bacteria (46–48). MGEs (mainly as plasmids and as integrative and conjugative elements) play a vital role in HGT and the coselection of ARGs and VFs in bacteria (30, 49, 50). ARGs and VFs can be disseminated extensively when equipped on the proper transfer machinery and efficient vehicles in gene shuffling, which could promote the evolution of microbe groups under environmental stress (51, 52). Sequence and functional genome analysis in previous studies revealed that many resistance and virulence genes in major pathogens have been obtained by horizontal transfer during evolution (53–55). This has caused serious outbreaks of superbugs in hospital and communal settings. Recently, an outbreak of carbapenem-resistant *Enterobacteriaceae* was attributed to the horizontal transfer of Klebsiella pneumoniae carbapenemase-encoding genes between strains and species by a conjugative plasmid (56). Lessons learned from transmissible ARGs and VFs in pathogens may lead to the development of antibiotic management strategies to control resistance and clinical outbreaks.

The cotransfer vehicles of all nearest ARG-VF couples were investigated for the strength of transfer preference for transposons, integrons, and plasmids (Fig. 4e). We found that a broad spectrum of ARG-VF couples could be cotransferred by the three genic vehicles. There could be a possible mechanism to help transposons and plasmids cotransfer certain gene couples. Transposons were more likely to transfer gene couples, such as bacitracin-toxins, mupirocin-antiphagocytosis, and aminoglycoside-adherence, while plasmids were much more likely to transfer gene couples of multidrug-secretion systems (57). No marked preference was observed in integrons containing a variety of functional gene cassettes. Of note, the ARG-VF couples transferred by high mobility potential MGEs, such as broad-host-range plasmids and class 1 integrons, could readily spread across species or even phylum boundaries (58).

**FIG 5:**
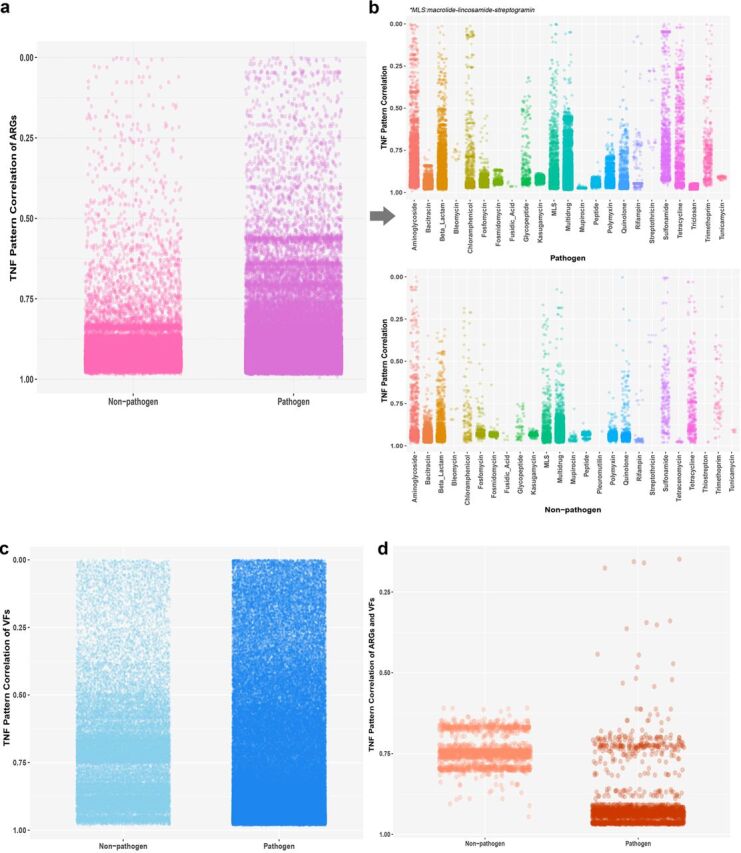
TNF pattern correlation between an ARG/VF-carrying fragment and its global genome sequence in pathogen and nonpathogen groups. (a and b) TNF pattern correlation profiles of fragments with ARGs; (c) TNF correlation profile of fragments with VFs; (d) TNF correlation profile of fragments with both ARGs and VFs.

To further evaluate the striking mobility of ARGs and VFs, the exogenously originated ARGs and VFs were identified across the large-scale genome collection (especially in human pathogens) (Fig. 5). Not surprisingly, compared to nonpathogens, the pairwise genomic comparisons showed more ARG/VF-carrying windows in human pathogens, in which the tetranucleotide frequency (TNF) signature substantially deviated from the genome-wide TNF pattern (correlation coefficient of <0.5). This indicated a higher incidence of exogenous acquisition of ARGs and VFs in human pathogens. The exogenous acquisition of VFs was also apparent in nonpathogens (Fig. 5c), which revealed the strong transfer ability of VFs. More interestingly, a clear bias in the distribution of exogenous fragments was detected in ARG-carrying windows (Fig. 5b). More than 70% of exogenous fragments were composed of genes conferring resistance against aminoglycoside, tetracycline, sulfonamide, and beta-lactam. This pattern corresponded with the mass consumption and long-period use of these common antibiotics in humans and animals (59, 60). Notably, a relatively higher incidence of exogenous fragments carrying glycopeptide resistance genes was observed in human pathogens. In view of the fact that glycopeptide (including vancomycin, teicoplanin, and telavancin) resistance genes are likely endemic components in nonpathogens, recent acquisition of these genes was regarded as being derived from environmental sources. Vancomycin, as the last line drug to defend against Gram-positive bacteria, such as Staphylococcus aureus, Streptococcus pneumoniae, and *Enterococcus*, has been prudently prescribed to treat infections caused by pan-drug-resistant pathogens over the past several decades (61, 62). Previous studies verified that a number of vancomycin resistance genes could be detected in swine manure samples (17), human feces samples (63), and even permafrost sediments that were segregated from anthropogenic activities (64), indicating that human pathogens have rapidly adapted to newly introduced antibiotics under infectious treatment. The majority of exogenous ARG/VF-carrying fragments (>60%) were identified from clinically important pathogen species of Acinetobacter baumannii, Escherichia coli, Klebsiella pneumoniae, and Salmonella enterica, in which HGT plays an important role in the development of resistance (28, 65, 66).

### Conclusions.

In this study, we first bonded a large-scale complete genome data set with powerful genetic analysis to investigate the ARG-VF coexistence in the background of microbes from multiple species and hosts. Our results revealed the diversity of ARG-VF coexistence in genomes from distinct pathogenicities and habitats, in which ARGs and VFs were found to have a strong correlation and an intimate genetic linkage in pathogen species and human/animal habitats. ARGs and VFs with high cotransfer potential were speculated to blur ecological boundaries. Moreover, we revealed the significant role of HGT in shaping resistome and pathogenicities of human commensal bacteria. In summary, the relationship between ARG and VF evaluated in this study may depend on four main factors: the bacterial species involved, potential pathogenicity, the habitats and hosts, and genetic transmission. Although this allowed us to obtain a comprehensive view of ARG-VF coexistence, we appreciate that the data set is likely affected by anthropogenic sampling biases, which are overrepresented in samples from regions with active surveillance and research foundations. Nevertheless, we believe our study highlights the potential risk of ARG-VF coexistence at both clinical and environmental levels. We provide a road map and a bioinformatics method for future gene correlation researches. We also hope that the coexistence relationships revealed by this study will be further validated using other approaches.

## MATERIALS AND METHODS

### Complete genome collection and data source retrieval.

To provide a comprehensive description of ARG-VF coexistence in bacteria, we randomly collected a large data set of bacterial complete genomes (*n* = 9,070, covering a large diversity of bacteria) from the NCBI RefSeq genome database (ftp://ftp.ncbi.nlm.nih.gov/genomes/refseq/bacteria) in March 2018 according to the proportions of different species in the database (considering the nonuniformity of data collection, the following analyses have eliminated the impact of sample size as much as possible). Taxonomic information of the downloaded data set was retrieved from the NCBI taxonomy database. The complete list of genomes is summarized in Table S1 in the supplemental material. The coding sequence (CDS) and their genetic location information were obtained to generate a downstream analysis. The habitat information was searched from the IMG database (https://img.jgi.doe.gov/) using the corresponding metadata (March 2018) by merging the fields of “habitat,” “ecology,” and “isolation” (Table S2). The potential pathogenicity of genome collections was obtained by mapping their taxonomic information with a published database covering all confirmed and potential species of human pathogens (67, 68).

10.1128/mSystems.00821-19.9 TABLE S1 Complete list of the genomes used in this study. Download Table S1, XLS file, 3.7 MB. Copyright © 2020 Pan et al. 2020 Pan et al. This content is distributed under the terms of the Creative Commons Attribution 4.0 International license.

10.1128/mSystems.00821-19.10 TABLE S2 Habitats listed in the IMG genome and the habitat metadata used in this study. Download Table S2, XLSX file, 0.01 MB. Copyright © 2020 Pan et al. 2020 Pan et al. This content is distributed under the terms of the Creative Commons Attribution 4.0 International license.

### ARG, VF, and MGE retrieval.

The DeepARG database (merging the nonredundant data of the three major databases: CARD, ARDB, and UniProt) and SARG database (containing a hierarchical structure of type–subtype–reference sequence) were used to identify and quantify a broad-spectrum of ARGs (69, 70). The VFDB (containing cumulative information of VFs for the most important bacterial pathogens, virulence-associated genes, protein structural features, functions, mechanisms, and important literatures) and Victors database (including virulence factors for many different microbes that are pathogenic to animals and humans [http://www.phidias.us/victors/]) were used to detect VFs (71). All the CDS of downloaded genomes were extracted to identify ARGs and VFs by searching against the above database. The CDS were assigned an E value cutoff of 1e–5 against the database using BLASTP. CDS were annotated as ARGs and VFs when their best hit had ≥90% identity and ≥80% query coverage of the reference sequences (72). To explore the moving ability and heritable stability of DNA fragments, MGEs were identified by string matches to one of the following keywords in the gene description: transposon, integron, conjugal, mobilization, recombination, and plasmid.

### Enrichment analysis.

After we filtered the low-quality BLASTP results, deduplication of ARG- and VF-like sequences was performed and then categorized into antibiotic and VF types, respectively (VFs were classified into 13 types according to the function and mechanism description in the databases). The enrichment of ARGs and VFs across the phylogenies, habitats, and pathogenicities were tested by the Fisher exact test, and the *P* value was further adjusted by the Benjamini-Hochberg correction for multiple comparisons. Significant enrichment was defined as a *P* value of <0.01 and an odds ratio of >1.

### ARG and VF coexistence analysis.

Two indexes, including the average minimum distance and the incidence of encountering, were used to evaluate the coexistence relationship of ARGs and VFs on circular genomes (including plasmid sequences). The genomes carrying both ARGs and VFs were extracted for the coexistence analysis. Next, the average minimum distances [MetAmin(bp)] were calculated on each genome as the sum of distances of the closest ARG from each VF divided by the number of VFs (equation 1 in Text S1 in the supplemental material). To calculate the incidence of gene encountering, we counted the number of ARGs within the assigned distance (1 to 100 kbp, by 1 kbp) from every VF in each genome, and the raw counts were then averaged in a given genome category (equation 2 in Text S1 in the supplemental material). The pathogen species, which were filtered by a standard of total ARG and VF numbers of ≥5 per genome and genome numbers of ≥5 per species, were considered to have high ARG and VF coexistence potential. To assess the diversity of coexistence patterns, the Shannon index was calculated by the number of unique ARG subtypes that were adjacent to VFs along a given distance (1 to 500 kbp) in each genome. The nearest ARG types of each VF type were summed up. Next, the MetAmin between ARGs/VFs and MGEs were calculated to estimate the gene cotransfer potential on the genome. The nearest ARG and VF from each MGE were deemed as an ARG-VF gene type pair (e.g., bacitracin-toxins) to analyze the cotransfer preference.

10.1128/mSystems.00821-19.8 TEXT S1 Calculation of the two distance indexes for gene coexistence. Equation 1 is used to calculate the average minimum distance [MetAmin(bp)] between genes A and B; equation 2 is used to calculate the incidence of encountering gene B within a given distance from gene A. Download Text S1, DOC file, 0.03 MB. Copyright © 2020 Pan et al. 2020 Pan et al. This content is distributed under the terms of the Creative Commons Attribution 4.0 International license.

### Cluster analysis.

A count matrix of the nearest ARG-VF pairs in each ARG-VF type (e.g., bacitracin-toxins) was created for each genome to compare the underlying coexistence structures among the different genome categories. The count matrix was used to calculate the Bray-Curtis distance matrix, and a PCoA was then performed using R with package vegan. The coexistence structure in different categories of genome clusters was based on a *P* value of <0.001 calculated by using ANOSIM. The biplot position was calculated by the weighted average of the coordinate position of all genomes in the PCoA, where the weight was the abundance of the ARG-VF type in every genome and was plotted in a dimensional space using the ggplot2 package in R.

To visualize the coexistence structure in important clinical pathogens, eight major pathogen species (Klebsiella pneumoniae, Acinetobacter baumannii, Staphylococcus aureus, Pseudomonas aeruginosa, Escherichia coli, Salmonella enterica, Enterococcus faecalis, and Enterococcus faecium) were analyzed through a network interface by counting their nearest ARG-VF types. A preliminary filter was implemented to remove those poorly represented ARG-VF pairs and reduce the artificial bias (those ARG-VF pairs were filtered, if the relative abundances were <0.01). The robust pairwise correlation of the ARG-VF pairs and the pathogen species mentioned above formed their coexistence network. The network and modularity classes were explored and visualized using the interactive platform of Gephi (73).

### Tetranucleotide signature analysis.

Tetranucleotide frequency (TNF) signatures contain a species-specific symbol (74) and have been shown to be a good compromise between computational calculation power and a pronounced phylogenetic signal (75). TNF signatures are widely used as an alignment-free genomic similarity index to depict the degree of sequence homology (76–78). To further evaluate the gene mobility and retrieve the exogenous ARGs and VFs, genome-wide TNF signatures were calculated (in forward and reverse strand) for each genome using the Biostrings package in R. By setting sliding window of 5 kbp (step = 1 kbp), TNF signatures were calculated across the whole length of each genome. Based on the TNF content comprising the whole genome and all the windows of each genome, the Pearson’s correlation coefficient (*r*^2^) was calculated between TNF counts [in terms of log(1+counts)] in each window and that of the corresponding whole genome to obtain the deviation of TNF in each window from the global pattern (78). Pearson correlation coefficients of the windows containing ARG were outlined based on the ARG type they carried.

### Data availability.

The data set supporting the conclusions of this article is included within the article and in the supplemental material.
