# Supplementary figures and images for: Coexistence of Antibiotic Resistance Genes and Virulence Factors Deciphered by Large-Scale Complete Genome Analysis
Source: mSystems. 2020 Jun 2;5(3):e00821-19. doi: 10.1128/mSystems.00821-19 (PMC8534731; doi:10.1128/mSystems.00821-19)

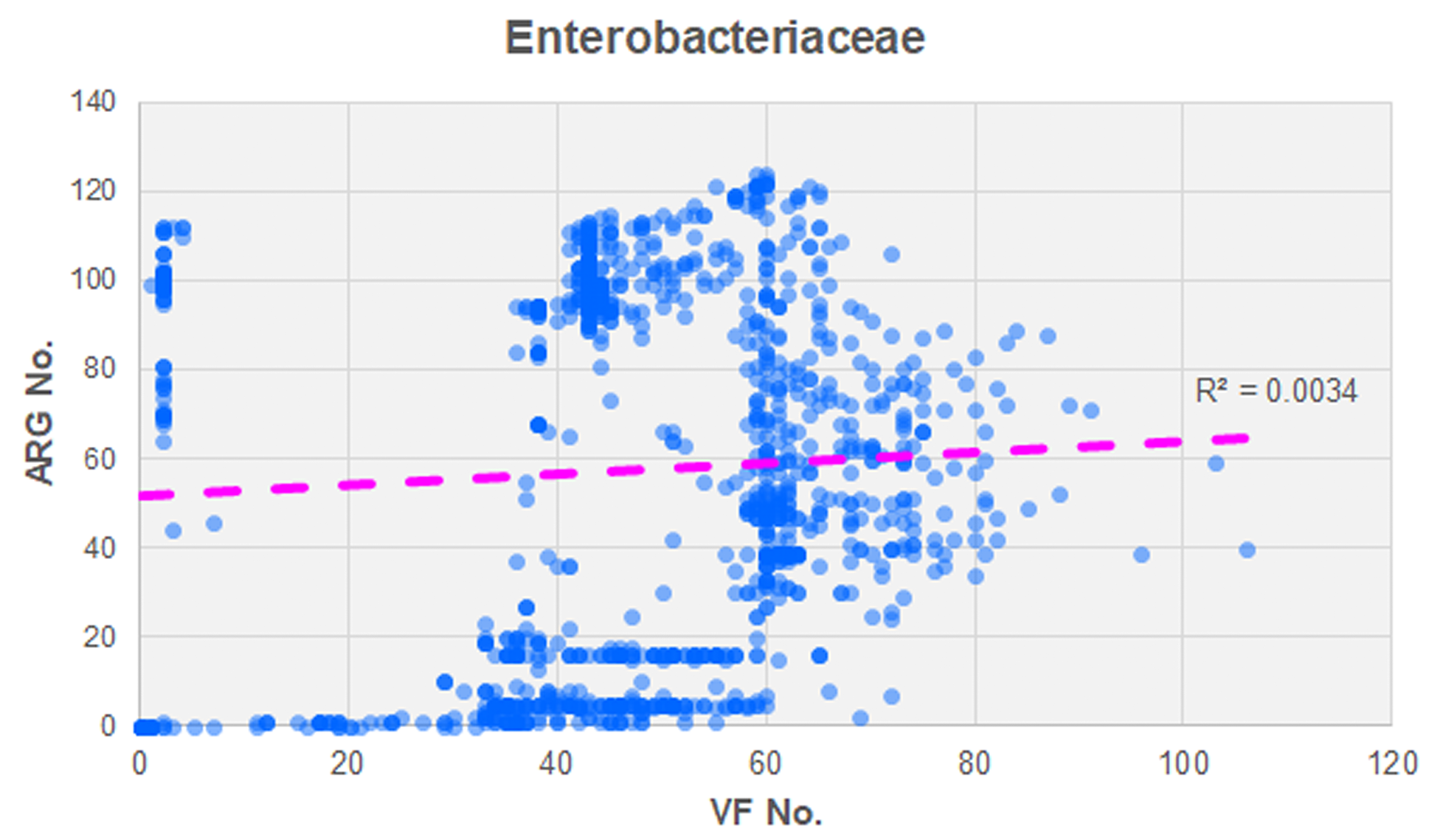

Supplement: FIG S2 [file msystems.00821-19-sf002.tif]

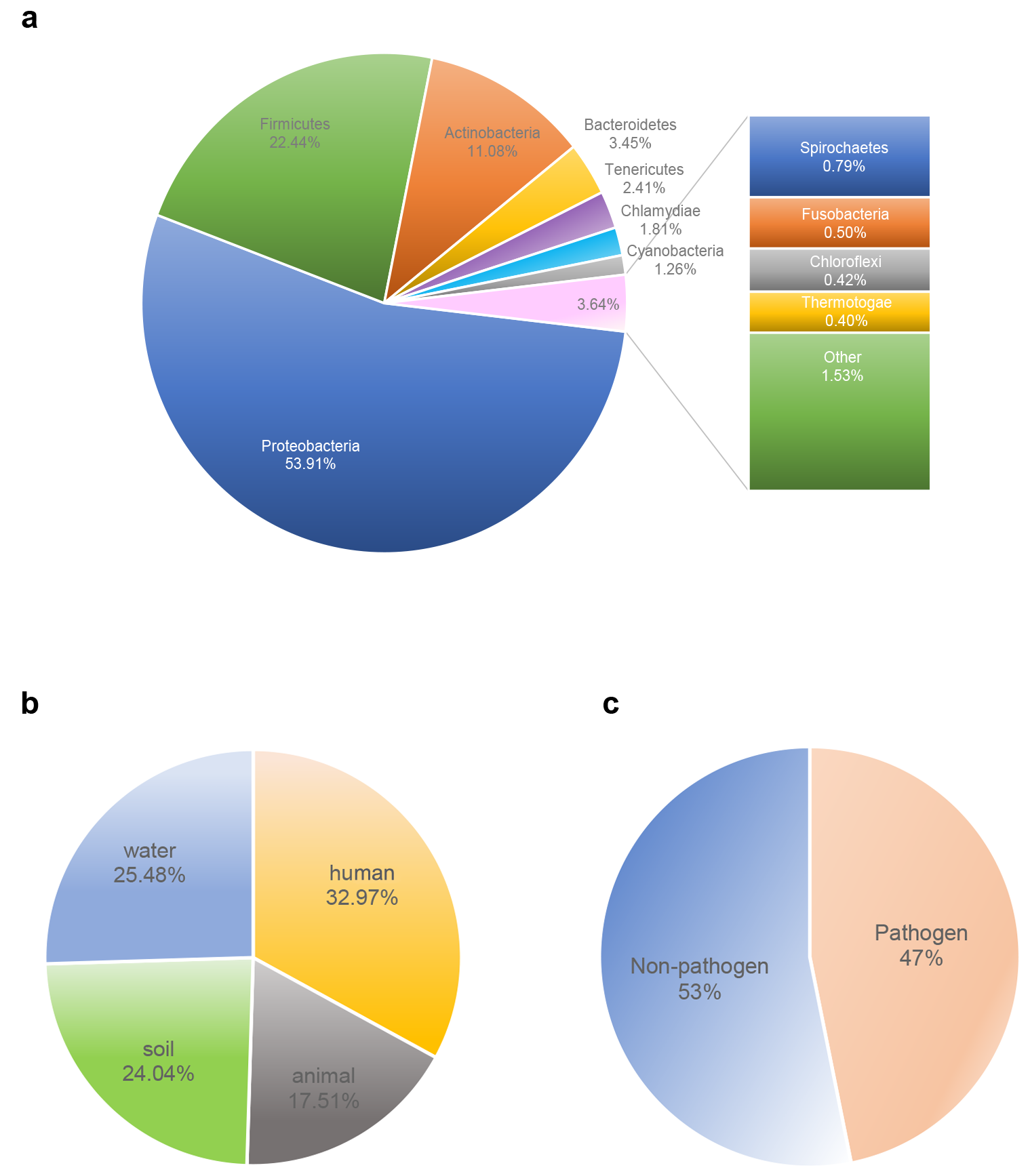

Supplement: FIG S3 [file msystems.00821-19-sf003.tif]

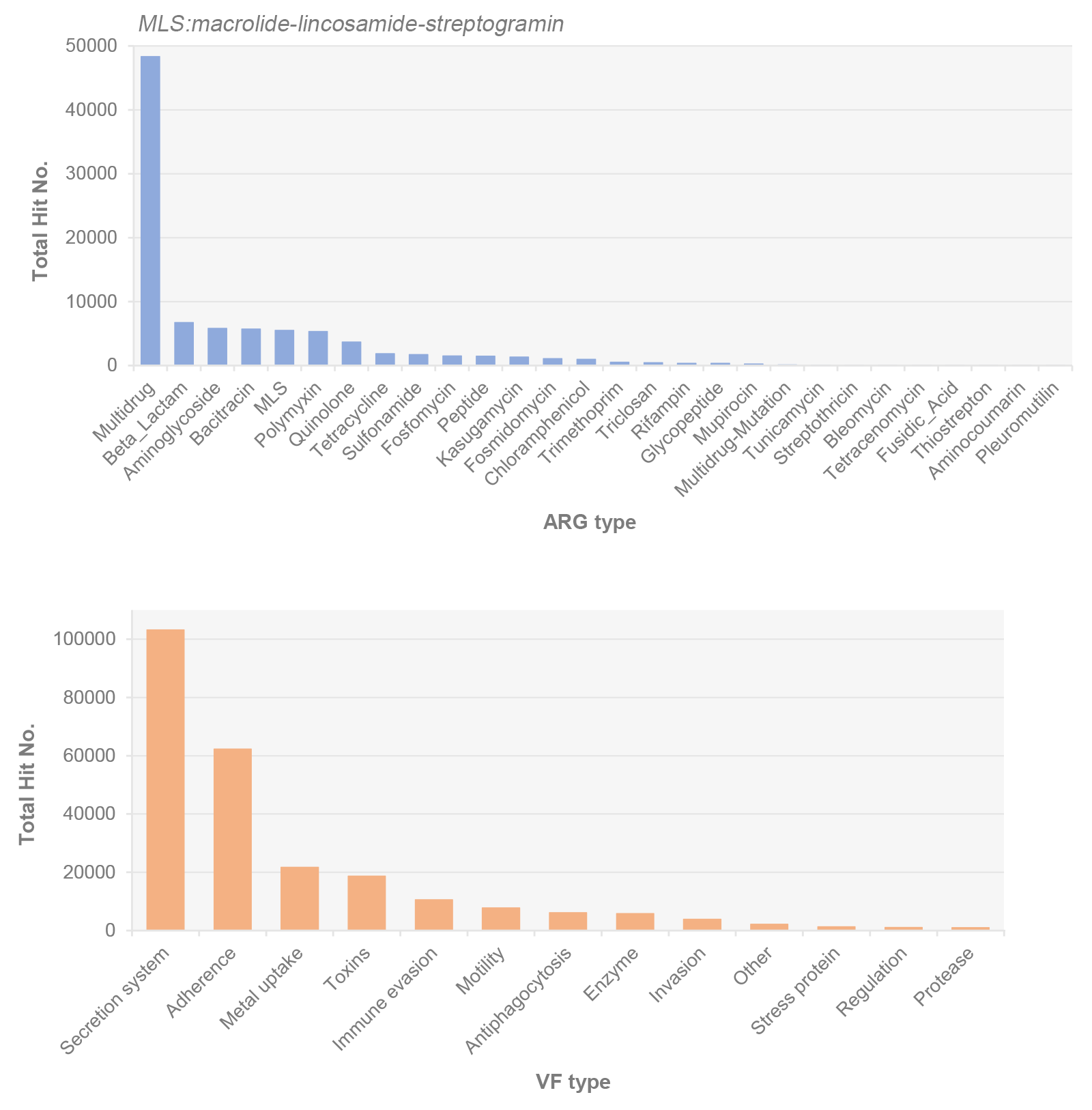

Supplement: FIG S4 [file msystems.00821-19-sf004.tif]

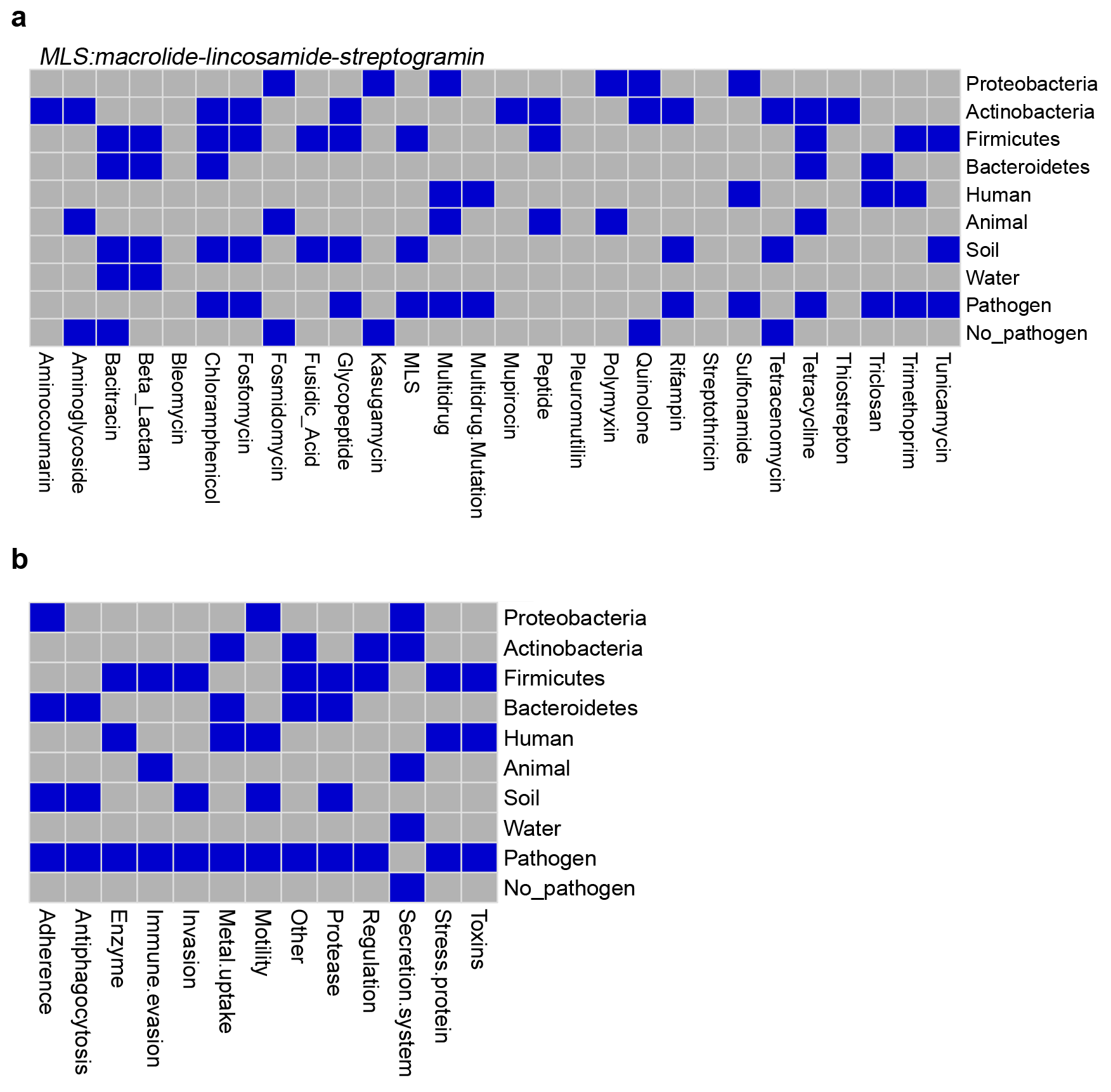

Supplement: FIG S5 [file msystems.00821-19-sf005.tif]

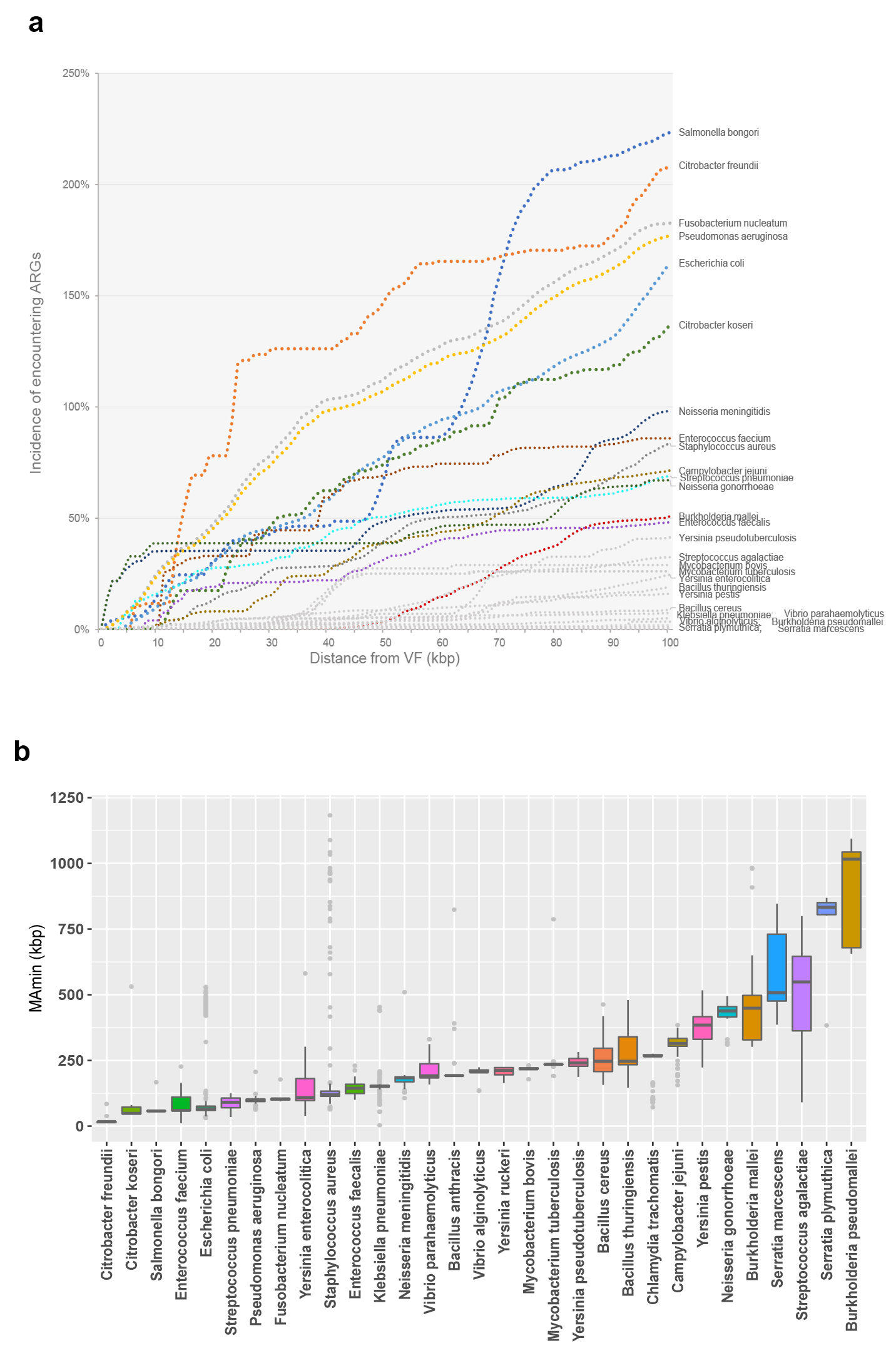

Supplement: FIG S6 [file msystems.00821-19-sf006.tif]

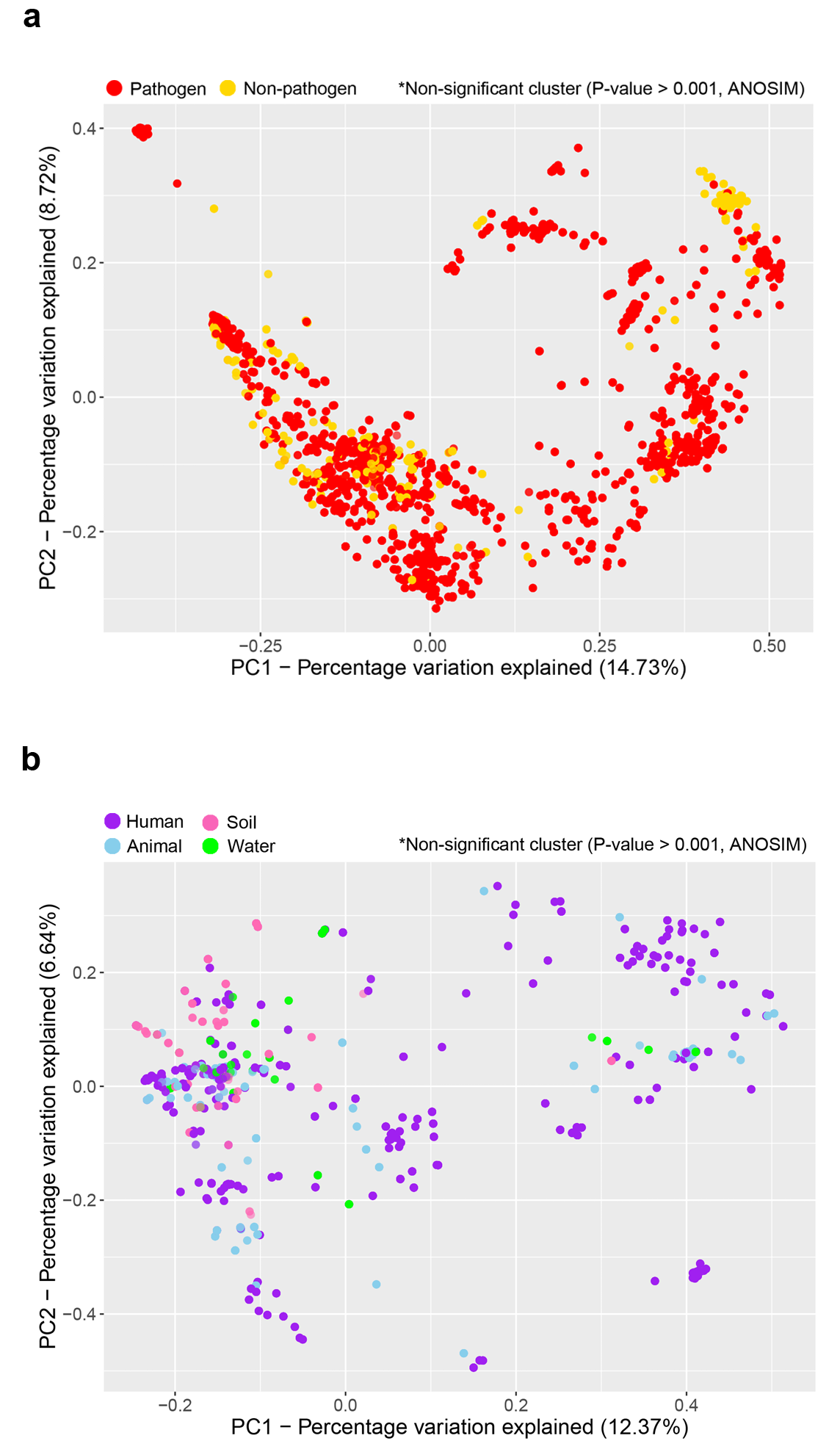

Supplement: FIG S7 [file msystems.00821-19-sf007.tif]
