# Supplementary material for: Coexistence of Antibiotic Resistance Genes and Virulence Factors Deciphered by Large-Scale Complete Genome Analysis
Source: mSystems. 2020 Jun 2;5(3):e00821-19. doi: 10.1128/mSystems.00821-19 (PMC8534731; doi:10.1128/mSystems.00821-19)
Supplement: TEXT S1 [file msystems.00821-19-s0001.doc]

# Supplementary Methods

**Calculation of the two distance indexes for gene coexistence**

Gene coexistence indexes for gene A driven co-selection of gene B：

- N: copy number of gene A.

- M: number of gene B within a given distance from gene A.

Equation (1): Average minimum distance (MetAmin(bp)) between gene A and B:

Equation (2): Incidence of encountering gene B within a given distance from gene A: ×100%

For a group of genomes (No.of genomes = K):

Incidence of encountering gene B within a given distance from gene A:
